# Comparative analysis of the efficacy of UBE-PLIF versus conventional PLIF in the treatment of L4-5 degenerative spondylolisthesis

**DOI:** 10.1186/s13018-025-06266-1

**Published:** 2025-09-26

**Authors:** Xinkai Luo, Yixi Wang, Yiqing Wu, Qiuyuan Huang, Zexi Wang, Zhen Wu, Xiaoyu Cai, Hailong Guo

**Affiliations:** 1 Department of Minimally Invasive Spine Surgery and Precision Orthopedics, The First Affiliated Hospital of Xinjiang Medical University, Urumqi, 830054 Xinjiang Uygur Autonomous Region China; 2 Department of Spine Surgery, The First Affiliated Hospital of Xinjiang Medical University, Urumqi, 830054 Xinjiang Uygur Autonomous Region China; 3 Department of Infectious Disease, The First Affiliated Hospital of Harbin Medical University, Harbin, 150000 China

**Keywords:** Lumbar spondylolisthesis, Unilateral dual-channel spinal endoscopic interbody fusion, Posterior lumbar interbody fusion

## Abstract

**Background:**

L4-5 lumbar degenerative spondylolisthesis is a common spinal disease in the middle-aged and elderly population, often accompanied by spinal stenosis and nerve root compression, which seriously affects the quality of life. Traditional posterior lumbar interbody fusion (PLIF) has been widely used in the treatment of such diseases, but it is more traumatic, has a longer recovery period, and has more complications. In recent years, Unilateral biportal endoscopic posterior lumbar Interbody Fusion (UBE-PLIF) has received attention as a minimally invasive treatment. However, the difference in efficacy between UBE-PLIF and PLIF remains to be further explored. This study aimed to compare the clinical outcomes and postoperative imaging changes between the two in the treatment of L4-5 degenerative spondylolisthesis and to provide a basis for clinical decision-making.

**Methods:**

Fifty-nine patients with L4-5 degenerative lumbar spondylolisthesis admitted between January 2021 and January 2024 were retrospectively analyzed in this study, including 28 in the UBE-PLIF group and 31 in the PLIF group. Baseline data (gender, age, history of hypertension/diabetes, BMI), major operative parameters (operative time, number of intraoperative fluoroscopies, postoperative drainage volume) and clinical assessments (low back pain/leg pain VAS score, ODI, SF-36) were collected, and a modified MacNab score was used for final follow-up. Imaging assessments included disc height, (DH), L4-5 segmental lumbar lordosis (SLL), lumbar lordosis (LL), and sagittal slip distance (SSD) preoperatively, at 3 days postoperatively, and the final follow-up, and were compared with the paravertebral muscle cross-sectional area (CSA), the paravertebral muscle fat infiltration (FI), Adjacent segment Pfirrmann grades, and vertebral fusion rate at the final follow-up.

**Results:**

Surgery was completed in both groups, with comparable baseline characteristics and significant postoperative symptom relief. The UBE-PLIF group had significantly less drainage but slightly longer operative time and more fluoroscopic exposures (*p* < 0.05). Both groups showed significant improvement in leg pain VAS, ODI, and SF-36 scores; however, low back pain VAS at 1 month was significantly lower in the UBE-PLIF group (*p* < 0.05). Final follow-up revealed no difference in modified MacNab “Excellent ”or “Good ”Rate (92.9% vs. 90.3%, *p* > 0.05). Radiologically, both groups demonstrated improved DH, SLL, LL, and SSD, with greater gains in SLL, LL, and SSD in the PLIF group (*p* < 0.05). Adjacent segment Pfirrmann grades showed no significant difference (*p* > 0.05). Although the proportion of Grade I fusion was higher in the UBE-PLIF group (64.3% vs. 54.8%), the difference was not statistically significant (*p* = 0.682). Notably, the UBE-PLIF group had superior paravertebral muscle CSA preservation and lower fat infiltration (*p* < 0.05). Complication rates were similar (7.1% vs. 12.9%, *p* = 0.465), with no major adverse outcomes after appropriate management.

**Conclusion:**

Both UBE-PLIF and conventional PLIF can achieve good clinical outcomes in the treatment of L4-5 degenerative lumbar spondylolisthesis. Compared with PLIF, UBE-PLIF has the minimally invasive advantages of less postoperative drainage, faster relief of low back pain, better protection of paravertebral muscles, and lower fat infiltration, and is also comparable to PLIF in terms of complication rate and fusion rate at the final follow-up, and adjacent segmental degeneration. Although PLIF was slightly superior in terms of the magnitude of improvement in some imaging metrics such as SLL, LL, and SSD, the clinical significance of the difference requires further investigation. Overall, UBE-PLIF provides a safe, effective, and less invasive surgical option for L4-5 degenerative spondylolisthesis.

## Background

L4–5 degenerative spondylolisthesis is a common spinal disorder, particularly affecting middle-aged and elderly individuals. It is characterized by anterior displacement of the vertebral body, often accompanied by spinal canal stenosis and nerve root compression, leading to low back pain, radicular symptoms, and motor dysfunction that significantly compromise patients’ quality of life and social functioning. With the accelerating ageing of the global population, the incidence of this condition has been rising steadily, making it one of the most prevalent and challenging degenerative spinal diseases encountered in clinical practice [1]. Although non-surgical approaches—such as pharmacologic therapy, physical rehabilitation, and lifestyle modification—may offer symptomatic relief in mild cases, surgical intervention remains the primary treatment option for patients with advanced disease, particularly those exhibiting neurological deficits or marked spinal instability [2].

Traditional posterior lumbar interbody fusion (PLIF), which entails discectomy, interbody fusion, and spinal stabilization via a posterior approach, has been widely employed in the management of degenerative lumbar spondylolisthesis. This technique effectively restores spinal stability, alleviates nerve root compression, and improves clinical symptoms. However, due to the extensive tissue dissection and the complexity of the involved anatomical structures, PLIF is associated with substantial surgical trauma, prolonged postoperative recovery, and a heightened risk of complications such as dural tears, nerve injury, and postoperative infection [2]. Moreover, its highly invasive nature may further compromise postoperative quality of life. These limitations have prompted increasing interest in the development of minimally invasive alternatives that aim to reduce intraoperative trauma, accelerate functional recovery, and lower complication rates [3].

In recent years, with the rapid advancement of endoscopic techniques, unilateral biportal endoscopic posterior lumbar interbody fusion (UBE-PLIF) has gained increasing attention as a novel minimally invasive surgical approach [4]. By utilizing a dual-channel endoscopic system, UBE-PLIF minimizes paraspinal muscle dissection and bony structure disruption commonly associated with traditional open procedures. This approach has been shown to significantly reduce intraoperative blood loss, shorten operative time, and lower the incidence of postoperative complications [5–7]. Furthermore, owing to its limited surgical trauma and accelerated postoperative recovery, UBE-PLIF is associated with reduced postoperative pain and more rapid improvement in functional outcomes compared with conventional PLIF [8].

However, despite the numerous theoretical advantages of UBE-PLIF, its comparative efficacy and radiological outcomes relative to conventional PLIF remain a subject of debate, and current evidence from systematic comparative studies is still limited. Therefore, this study aimed to evaluate and compare the clinical efficacy and postoperative imaging outcomes of UBE-PLIF and PLIF in the treatment of L4–5 degenerative lumbar spondylolisthesis by retrospectively analyzing data from patients treated between January 2021 and January 2024. By examining differences in multiple dimensions—including pain relief, functional recovery, complication rates, and radiological improvement—this study seeks to clarify the clinical advantages and limitations of UBE-PLIF as a minimally invasive surgical option and to provide a more robust basis for evidence-based surgical decision-making.

## Clinical data

### Case inclusion and exclusion criteria

Retrospective study. Collect the clinical data of L4-5 degenerative lumbar spondylolisthesis treated by UBE-PIF and PLIF from January 2021 to January 2024 in our hospital. Case inclusion criteria: [1] Lateral lumbar spine radiographs showed L4-5 Meyerding I and II degree lumbar spondylolisthesis; [2] Slip distance > 3 mm, regardless of X-ray hyperextension/hyperflexion position; [3] Patients with lumbar and bilaterally painful lower extremities; [4] Patients who were ineffective after receiving at least 12 weeks of conservative treatment; [5] Cobb < 10°; [6] Complete preoperative and postoperative imaging data patients; [7] patients with a follow-up period of at least more than 1 year;

Exclusion criteria: [1] Lateral X-ray of the lumbar spine showed Meyerding’s slip of degree III and above; [2] patients with combined isthmic fracture of L4-5 segment; [3] patients with a previous history of lumbar spine surgery; [4] Cobb ≥ 10°; [5] patients with combined spinal fracture, spinal infection, spinal tumour, or ankylosing spondylitis [6]. Loss of postoperative visits.

### General information of patients in the two groups

According to the inclusion and exclusion criteria, a total of 64 patients met the selection criteria for inclusion in the study and 5 were lost to follow-up. Among them, there were 2 cases in the UBE-PLIF group and 3 cases in the PLIF group. Age, gender, presence of hypertension/diabetes mellitus, body mass index (BMI), as well as preoperative, postoperative 3-day, 1-month, 6-month and final follow-up VAS scores for low back/leg pain, ODI, SF-36 and final follow-up modified MacNab scores, were collected in both groups, surgical parameters were collected in both groups, operative time, a number of intraoperative fluoroscopies, postoperative drainage volume, and imaging data were collected on the disc height (DH), L4-5 segmental lumbar lordosis (SLL), lumbar lordosis (LL), sagittal slip distance (SSD) before, at 3 days after surgery and at the final follow-up, and to compare the paravertebral muscle cross-sectional area (CSA), the paravertebral muscle fat infiltration (FI), Adjacent segment Pfirrmann grades and vertebral fusion rate at the final follow-up. All surgeries were performed by the same two senior spine surgeons with extensive surgical experience in both endoscopic and open procedures.

### Surgical methods

UBE-PLIF group: After induction of general anesthesia, the patient was positioned prone, and C-arm fluoroscopy was used to mark the junction of the L4 spinous process base and left L4 lamina, as well as the bilateral L4 and L5 pedicles. After sterile preparation and draping, two approximately 1 cm longitudinal incisions were made along the lateral border of the left pedicle projection—at the level of, and 1 cm above and below, the initial landmark. Soft tissue retractors were inserted to dissect part of the paraspinal muscles subperiosteally, establishing separate observation and working portals. Through these, an endoscope and surgical instruments were introduced. Under endoscopic visualization, soft tissues over the lamina were removed using a radiofrequency knife to expose the spinous process base, lower edge of the L4 lamina, facet joint, and upper edge of the L5 lamina. The inferior facet joint and lower margin of the L4 lamina were excised using a rongeur and burr to expose the ligamentum flavum insertion. The tip of the L5 superior articular process and its lamina were similarly removed to expose the contralateral ligamentum flavum, which was resected en bloc to expose the dura mater and left L5 nerve root. Hypertrophic bone and protruding nucleus pulposus compressing the neural element were carefully removed, and the nerve root canal was decompressed. The L5 nerve root and dura were gently retracted medially with a nerve root retractor to expose the disc annulus, which was incised, and the nucleus pulposus was removed. Sequential reaming (7–10 mm) prepared the disc space, and endplates were cleaned under endoscopic guidance. A mix of autologous and artificial bone was implanted and compacted into the disc space, followed by placement of a suitably sized cage, whose position was confirmed intraoperatively by fluoroscopy. A burr and rongeur were used to enlarge the L4 spinous process base and remove residual contralateral ligamentum flavum and bony overgrowth, ensuring relaxation of the opposite L5 nerve root. Working instruments were withdrawn, and four appropriately sized pedicle screws—bone cement–augmented screws were used in cases of severe preoperative osteoporosis—were placed bilaterally under fluoroscopic guidance, connected using two pre-bent rods, and tightened after vertebral reduction. Final fluoroscopy confirmed appropriate screw placement and vertebral alignment. The hemostasis was confirmed, a drainage tube was placed, and the incisions were closed in layers with sterile dressings applied.

PLIF group: After induction of general anesthesia, the patient was placed in the prone position, and the surgical field was prepared with routine disinfection and sterile draping. A midline incision of approximately 8 cm was made from the L4 to L5 spinous processes, and the paraspinal muscles were dissected subperiosteally to the lateral border of the facet joints. Following lamina retraction, the entry points for the L4 and L5 pedicle screws were exposed, and under fluoroscopic guidance, four appropriately sized imported titanium pedicle screws were inserted bilaterally; bone cement–augmented screws were used in cases of severe preoperative osteoporosis. Intervertebral segmental decompression at L4–L5 was then performed. The superior margins of the L4 and L5 laminae were carefully removed with a gun-shaped rongeur, the hypertrophied ligamentum flavum was excised, and the dural sac and nerve roots were fully decompressed to the neural foramina. The left L5 nerve root was gently retracted to expose the intervertebral disc; the nucleus pulposus was excised with a scalpel, and the remaining disc material was removed with nucleus pulposus forceps; the upper and lower endplates were then prepared to the subchondral bone. Two connecting rods and four nuts were installed, and reduction of the L4 vertebral body was achieved under fluoroscopic guidance before securing the construct. Autologous bone harvested during decompression was morselized and implanted into the L4–L5 disc space from the left side along with an appropriately sized interbody fusion cage. Both L4 and L5 nerve roots were re-examined, confirming marked relief of nerve tension and absence of residual compression, and final fluoroscopy demonstrated satisfactory reduction and optimal implant positioning. The wound was irrigated with copious saline, meticulous hemostasis was achieved, a drainage tube was placed, and the incision was closed in layers with a sterile dressing applied.

### Postoperative management and outcome evaluation

Postoperative management was standardized across both groups. Patients were instructed to remain on bed rest immediately after surgery, with appropriate administration of analgesics and prophylactic measures to prevent deep vein thrombosis. From postoperative days 1 to 3, patients were advised to wear a lumbar brace and gradually resume light activity, with drainage tubes removed once output had sufficiently decreased. Upon discharge, patients were instructed to avoid heavy lifting and excessive lumbar flexion, to continue wearing lumbar support for at least one month, and to engage in moderate exercises aimed at strengthening the paraspinal and lumbar musculature, with progressive increases in activity intensity over time.

Operative time, number of intraoperative fluoroscopy exposures, and postoperative drainage volume were recorded for both groups. Clinical outcomes were assessed using the visual analogue scale (VAS) for low back and leg pain, the Oswestry Disability Index (ODI), and the Short Form-36 (SF-36) at baseline, postoperative day 3, 1 month, 6 months, and the final follow-up. The modified MacNab criteria were used to evaluate surgical efficacy at the last follow-up. Radiographic assessments included adjacent segment Pfirrmann grades, paravertebral muscle cross-sectional area (CSA), and fat infiltration (FI) based on lumbar MRI at baseline and final follow-up, as well as disc height (DH), segmental lumbar lordosis at L4–5 (SLL), lumbar lordosis (LL), sagittal slip distance (SSD), and interbody fusion rate on lateral lumbar spine X-rays at baseline, 3 days postoperatively, and final follow-up.

**Fig. 1:**
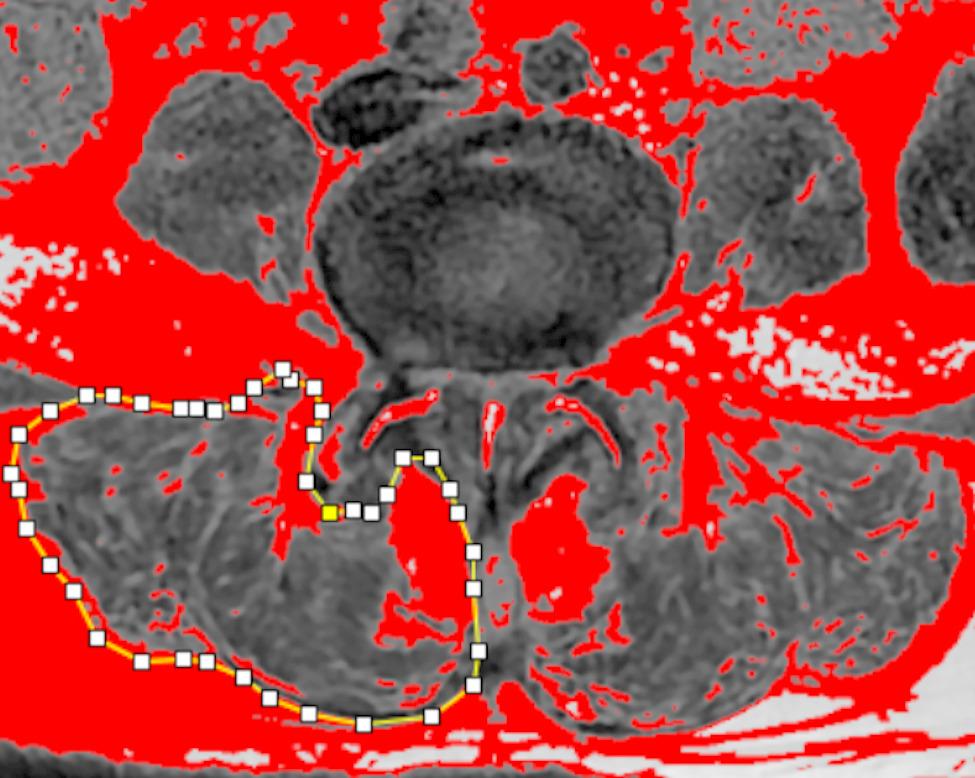
The image was processed using Image J software, with solid and dotted lines outlining the paravertebral muscle region. Fat infiltration (FI) of the paravertebral muscles was measured by applying the thresholding technique in Image J to T2-weighted MRI cross-sectional images of the lumbar spine, where red areas indicated fat infiltration. FI was then quantified for each paravertebral muscle region using the software tools

**Fig. 2:**
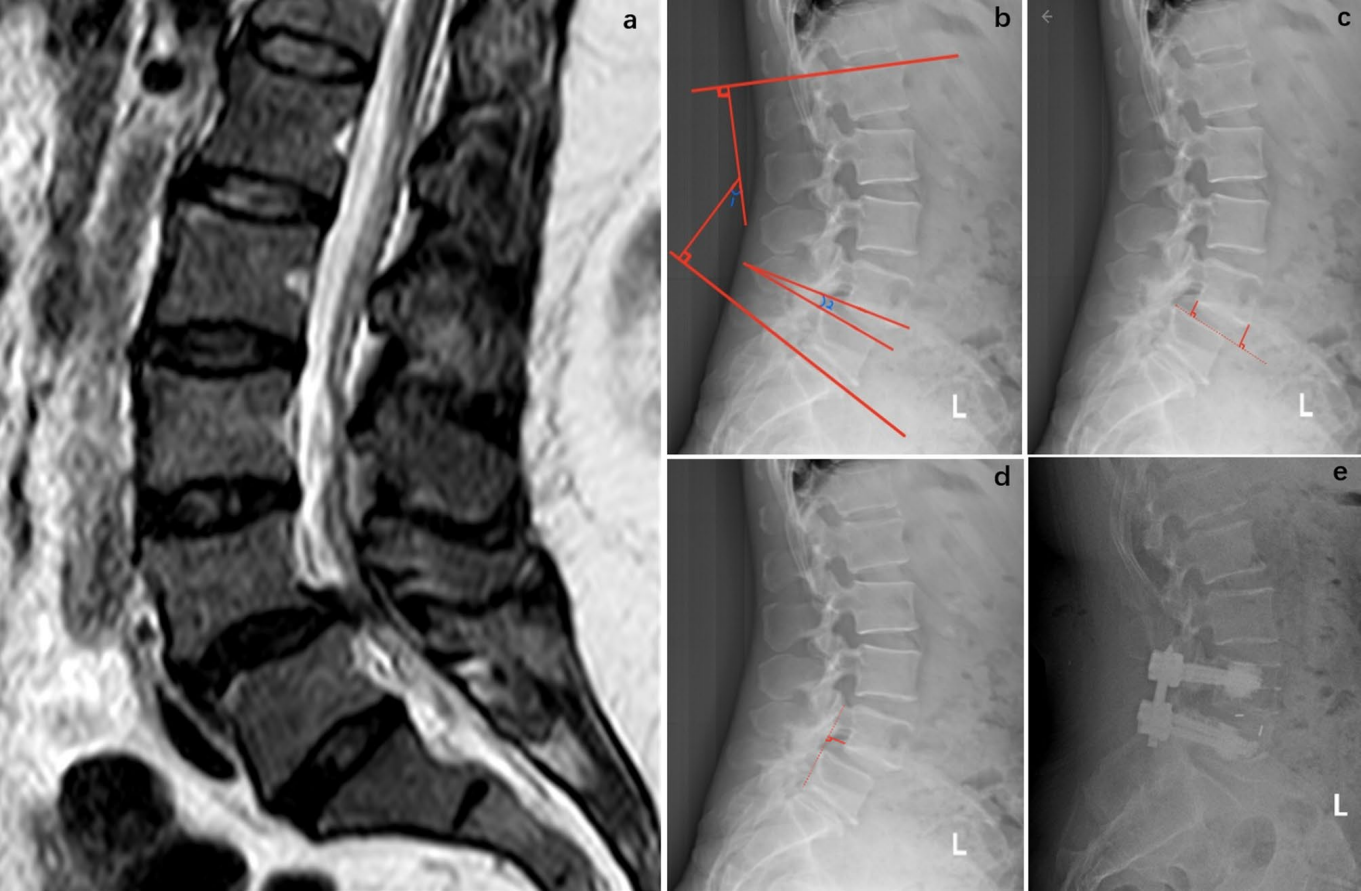
Figure “**a**” displays a midsagittal T2-weighted MRI image showing Pfirrmann grade 4 degeneration (marked disc degeneration) at both the L3–4 and L5–S1 levels. Figure “**b**” illustrates two angular measurements: the label 1 indicates overall lumbar lordosis (LL), measured via the Cobb angle between the superior endplate of L1 and that of S1; the label 2 denotes the segmental lumbar lordosis (SLL) at L4–5, measured using the Cobb angle between the inferior endplate of L4 and the superior endplate of L5. Figure “**c**” demonstrates the measurement of disc height (DH) at the L4–5 level. DH is calculated as the mean of the anterior and posterior disc heights, each indicated by a solid red line drawn perpendicularly between the superior and inferior endplates. Figure “**d**” shows the sagittal slip distance between L4 and L5. The solid red line highlights the horizontal displacement of the superior endplate of L4 relative to the posterior border of the superior endplate of L5. Figure “**e**” presents a postoperative lateral X-ray image, demonstrating solid arthrodesis consistent with Bridwell Grade I fusion, with clearly visible interbody implant and successful fusion

ImageJ software (NIH, USA) was used to analyze paravertebral muscle CSA and FI, with a grayscale threshold of 120 applied to quantify fat pixels within the muscle region, expressed as a percentage to reflect muscle atrophy. Detailed data are shown in Fig. 1.The Bridwell interbody fusion grading system was used to evaluate fusion outcomes at the final follow-up and is categorized into four grades [9]. Grade I represents complete fusion with continuous remodelling and trabecular bone formation, indicating solid osseous healing. Grade II refers to an intact graft without radiolucent lines, although remodelling or complete fusion has not yet occurred, suggesting ongoing incorporation. Grade III denotes an intact graft with radiolucencies at its superior and/or inferior interfaces, indicating partial or incomplete fusion. Grade IV was defined as a failure of spinal fusion, with signs of graft collapse or resorption and no radiographic evidence of solid bony union [10]. Measurements were performed using Surgimap software: LL was defined as the angle between the upper endplates of L1 and S1, SLL as the angle between the lower endplate of L4 and the upper endplate of L5, DH as the mean of anterior and posterior intervertebral disc heights, and SSD as the anteroposterior displacement between L4 and L5. Detailed data are shown in Fig. 2.

### Statistical methods

All statistical analyses were performed using SPSS version 27.0 (IBM Corp., Armonk, NY, USA). Categorical variables were presented as frequencies and percentages, with between-group comparisons conducted using the Chi-square test or Fisher’s exact test as appropriate. The normality of continuous variables was assessed using the Shapiro–Wilk test. Normally distributed data were expressed as mean ± standard deviation (SD) and compared using independent samples *t*-tests, whereas non-normally distributed data were expressed as the median and interquartile range (IQR) and analyzed using the Wilcoxon rank-sum test. For intragroup comparisons of normally distributed variables—including VAS scores for back and leg pain, ODI, SF-36, DH, LL, SLL, SSD, paravertebral muscle CSA and FI—one-way analysis of variance (ANOVA) was used, followed by post hoc comparisons using the least significant difference (LSD) test or Tamhane’s T2 test, depending on variance homogeneity. A two-tailed *p*-value < 0.05 was considered statistically significant.

## Results

### Surgical outcomes and Follow-up

Both groups completed the surgical procedures, with notable improvement in postoperative clinical symptoms. Clinical scale data were collected through outpatient visits or telephone follow-ups while imaging data were obtained during routine outpatient reviews. All patients were followed for a minimum of 12 months. Two patients in the UBE-PLIF group and three in the PLIF group were lost to follow-up.

### General information

There were no statistically significant differences between the UBE-PLIF and PLIF groups in terms of gender, age, body mass index (BMI), or the prevalence of hypertension and diabetes (*p* > 0.05). Compared with the PLIF group, the UBE-PLIF group had significantly lower postoperative drainage volume, although operative time and the number of intraoperative fluoroscopy exposures were slightly higher; these differences were statistically significant (*p* < 0.05). Detailed data are shown in Table 1.

**Table 1:** Comparison of preoperative characteristics and intraoperative parameters between the two groups

	UBE-PLIF(*n* = 28)	PLIF(*n* = 31)	t-value/χ2	*p*-value
Age	63.54 ± 6.62	62.23 ± 8.01	1.06	0.31
BMI	25.36 ± 2.81	25.26 ± 2.43	0.38	0.536
Female	15	18	0.12	0.73
Hypertension	11	15	0.49	0.48
Diabetes	9	12	0.277	0.59
**Pfirrmann Grading(L3-4)**				
Grade I	0	0	0.45	0.93
Grade II	3	2		
Grade III	6	6		
Grade IV	16	19		
Grade V	3	4		
**Pfirrmann Grading(L5-S1)**				
Grade I	0	0	0.29	0.86
Grade II	0	0		
Grade III	4	3		
Grade IV	18	21		
Grade V	6	7		
**Perioperative characteristics**				
operative time	187.89 ± 14.23	128.87 ± 20.96	7.99	<0.0001
IFE	28.32 ± 3.83	16.23 ± 1.71	27.77	<0.0001
PDV	57.54 ± 11.57	237.42 ± 33.53	19༎35	<0.0001

IFE: Number of intraoperative fluoroscopy exposures. PDV: Postoperative drainage volume

### Clinical scale outcomes

**Fig. 3:**
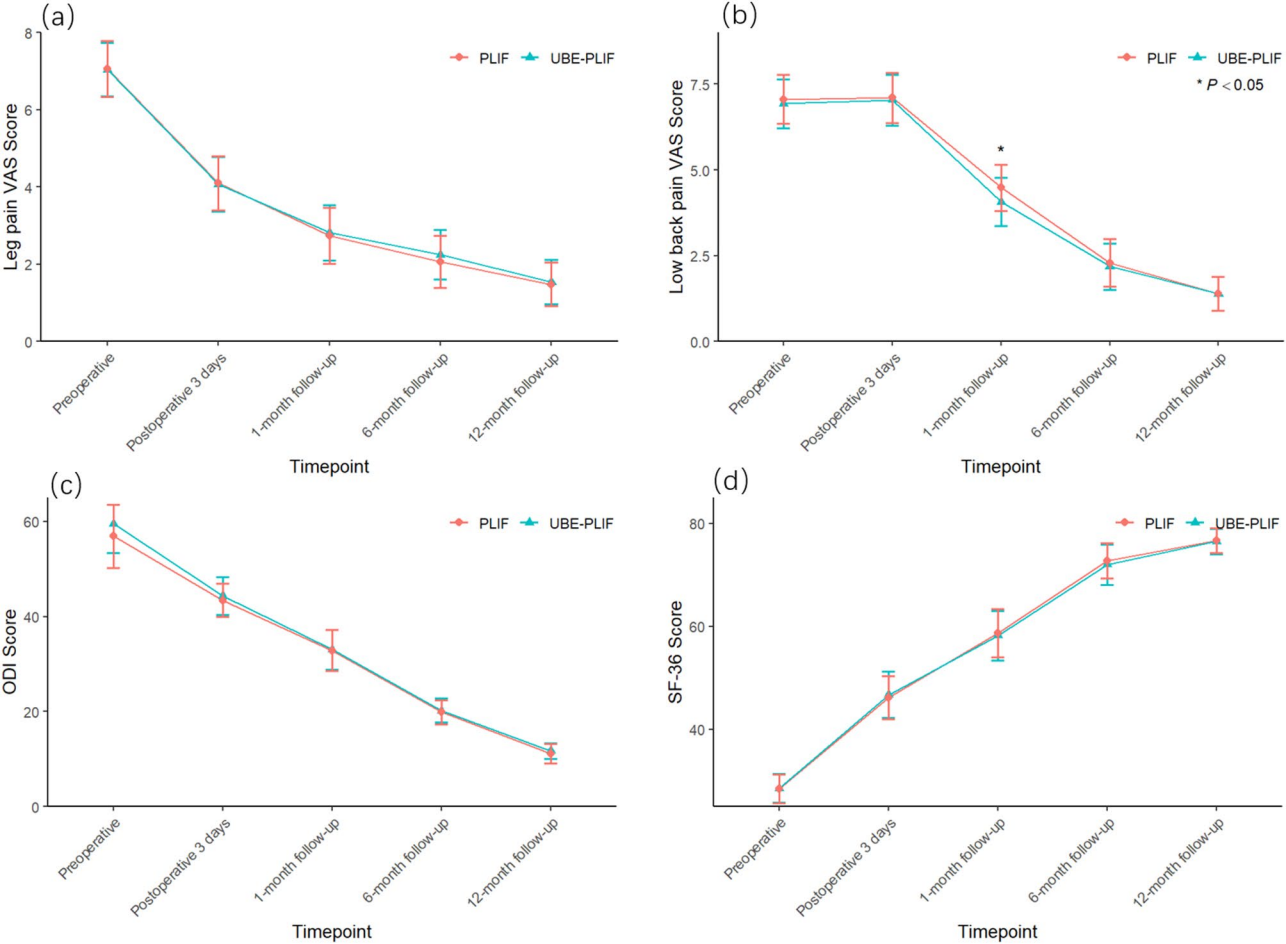
Comparison of Preoperative and Postoperative Scale Scores Between the Two Groups in the Figure (**a**)–(**b**) Visual Analog Scale (VAS) scores for leg pain and low back pain, respectively, before surgery and at postoperative follow-up. (**c**) Oswestry Disability Index (ODI) score before surgery and at follow-up. (**d**) 36-Item Short Form Survey (SF-36) score before surgery and at follow-up

Both groups showed significant improvement in low back and leg pain symptoms following surgery. Within-group analysis revealed that except for low back pain VAS scores on postoperative day 3—which did not differ significantly from preoperative values (*p* > 0.05)—VAS scores at all other postoperative time points improved significantly (*p* < 0.05). The between-group comparison showed that the UBE-PLIF group had significantly lower low back pain VAS scores at 1 month postoperatively compared to the PLIF group (*p* < 0.05), while no significant differences were observed at other time points (*p* > 0.05). ODI and SF-36 scores improved significantly in both groups at all postoperative time points compared to preoperative values (*p* < 0.05), with no significant differences between groups (*p* > 0.05). At final follow-up, the rate of “excellent” or “good” outcomes based on the modified MacNab criteria was 92.9% (26/28) in the UBE-PLIF group and 90.3% (28/31) in the PLIF group, with no statistically significant difference (*p* > 0.05). Detailed data are shown in Fig. 3; Table 2.

**Table 2:** Comparison of preoperative and Follow-up clinical data between the two groups

	UBE-PLIF(*n* = 28)	PLIF(*n* = 31)	t-value/χ2	*p*-value
**Low back pain VAS**				
Preoperative	6.93 ± 0.71	7.06 ± 0.72	-0.72	0.47
Postoperative 3 days	7.04 ± 0.74	7.1 ± 0.74	-0.31	0.75
1-month follow-up	4.07 ± 0.71	4.48 ± 0.67	-2.27	0.02
6-month follow-up	2.18 ± 0.67	2.29 ± 0.69	-0.62	0.53
12-month follow-up	1.39 ± 0.49	1.39 ± 0.49	0.05	0.96
F	421.19	476.28		
*P*	<0.0001	<0.0001		
**Leg pain VAS**				
Preoperative	7.04 ± 0.69	7.06 ± 0.72	-0.15	0.87
Postoperative 3 days	4.07 ± 0.71	4.1 ± 0.7	-0.13	0.89
1-month follow-up	2.82 ± 0.72	2.74 ± 0.72	0.42	0.67
6-month follow-up	2.25 ± 0.64	2.06 ± 0.68	1.07	0.28
12-month follow-up	1.54 ± 0.57	1.48 ± 0.57	0.34	0.73
F	289.02	327.57		
*P*	< 0.0001	<0.0001		
**ODI**				
Preoperative	59.57 ± 6.17	56.90 ± 6.66	1.59	0.16
Postoperative 3 days	44.36 ± 3.96	43.45 ± 3.50	0.93	0.35
1-month follow-up	33.04 ± 4.21	32.87 ± 4.28	0.15	0.88
6-month follow-up	20.21 ± 2.54	19.87 ± 2.51	0.52	0.61
12-month follow-up	11.75 ± 1.64	11.16 ± 2.04	1.21	0.23
F	633.57	603.5		
*P*	< 0.0001	<0.0001		
**SF-36**				
Preoperative	28.57 ± 2.84	28.45 ± 2.83	0.16	0.87
Postoperative 3 days	46.71 ± 4.5	46.16 ± 4.15	0.49	0.62
1-month follow-up	58.25 ± 4.82	58.68 ± 4.71	-0.34	0.73
6-month follow-up	72.04 ± 3.9	72.74 ± 3.43	-0.73	0.46
12-month follow-up	76.54 ± 2.47	76.68 ± 2.44	-0.22	0.83
F	729.25	932.01		
*P*	< 0.0001	<0.0001		
**Modified Macnab**				
Excellent	21	21		
Good	5	7		
Fair	2	3	0.38	0.826
Poor	0	0		
EGR	92.86%	90.33%		

EGR: Excellent and Good Rate

### Imaging data

**Fig. 4:**
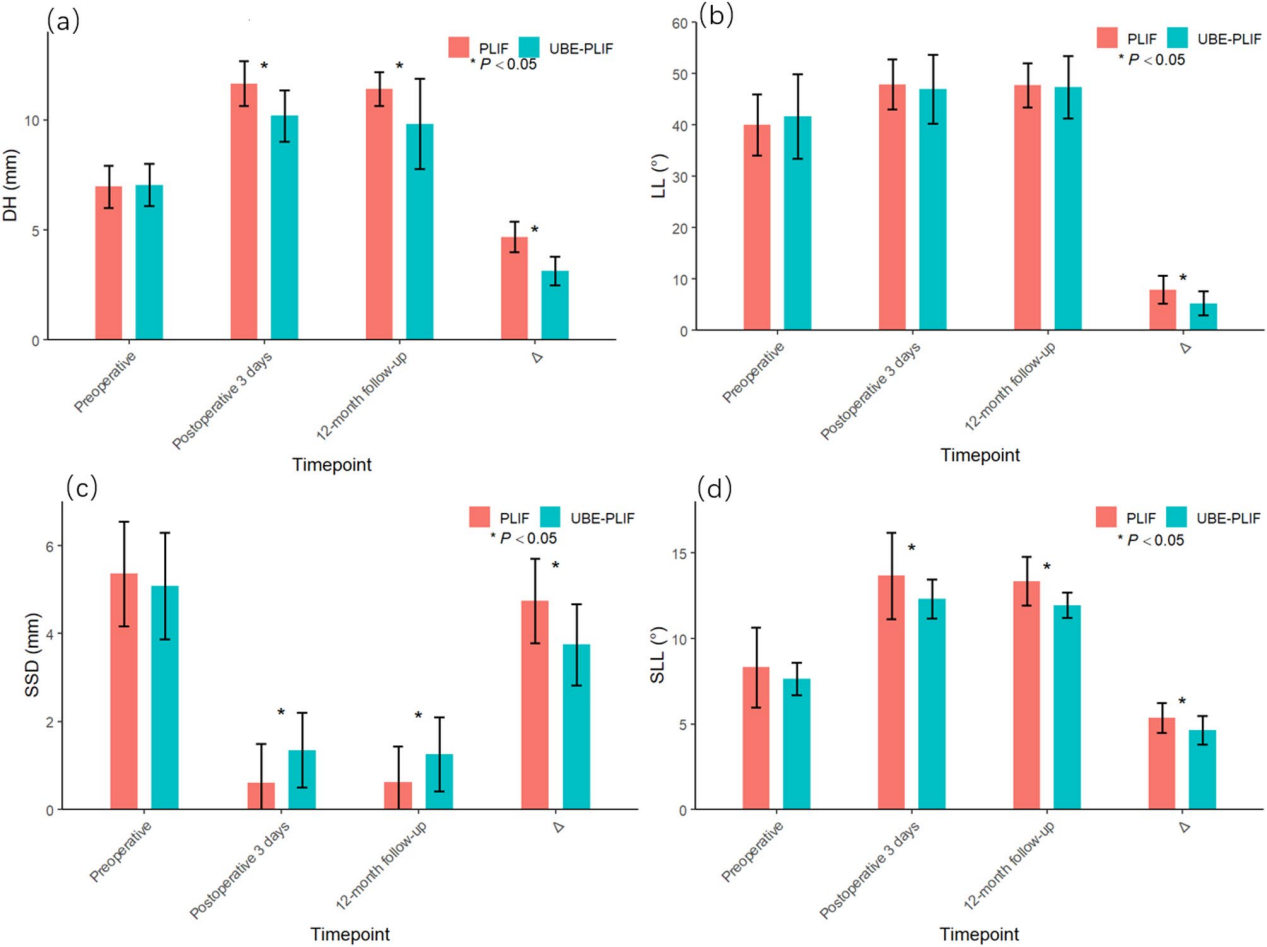
Comparison of Preoperative and Follow-up Imaging Data Between the Two Groups DH: Disc Height. LL: Lumbar Lordosis. SLL: Segmental Lumbar Lordosis. SSD: Sagittal Slip Distance Δ: Difference between postoperative day 3 and preoperative values. (a–d) Comparisons of disc-related sagittal radiographic parameters—including DH, LL, SSD, and SLL—between the two groups preoperatively and at postoperative follow-up

Postoperative imaging revealed significant improvements in lumbar lordosis (LL), L4-5 segmental lumbar lordosis (SLL), sagittal slip distance (SSD), and disc height (DH) in both groups compared to preoperative values (*p* < 0.05). Although both groups demonstrated statistically significant radiographic improvements, the PLIF group showed greater enhancements in LL, SLL, and SSD. However, this group also exhibited a significantly greater reduction in paravertebral muscle cross-sectional area (CSA) and higher fat infiltration (FI) compared to the UBE-PLIF group (*p* < 0.05), suggesting greater soft tissue disruption. At the final follow-up, no statistically significant difference was observed between the groups in the Pfirrmann grading of adjacent intervertebral discs (*p* > 0.05). Detailed data are shown in Fig. 4; Table 3.

**Table 3:** Comparison of preoperative and Follow-up imaging data between the two groups

	UBE-PLIF(*n* = 28)	PLIF(*n* = 31)	t-value/χ2	*p*-value
**DH(mm)**				
Preoperative	7.04 ± 0.96	6.97 ± 0.97	0.28	0.777
Postoperative 3 days	10.19 ± 1.17	11.65 ± 1.02	-5.11	<0.0001
12-month follow-up	9.83 ± 2.05	11.41 ± 0.78	-3.39	<0.0001
Δ	3.14 ± 0.65	4.68 ± 0.69	-8.85	<0.0001
F	171.61	471.58		
*P*	0.0001	<0.0001		
**SLL(°)**				
Preoperative	7.65 ± 0.95	8.31 ± 2.33	-1.37	0.147
Postoperative 3 days	12.3 ± 1.14	13.68 ± 2.53	-2.64	0.011
12-month follow-up	11.95 ± 0.75	13.35 ± 1.42	-4.64	<0.0001
Δ	4.64 ± 0.85	5.37 ± 0.86	-3.22	0.002
F	425.77	137.63		
*P*	< 0.0001	<0.0001		
**LL(°)**				
Preoperative	41.71 ± 8.21	40.03 ± 5.98	0.91	0.36
Postoperative 3 days	46.97 ± 6.71	47.96 ± 4.85	-0.65	0.51
12-month follow-up	47.35 ± 6.06	47.76 ± 4.28	-0.3	0.76
Δ	5.25 ± 2.34	7.93 ± 2.73	-4.01	<0.0001
F	295.49	526.49		
*P*	<0.0001	<0.0001		
**SSD(mm)**				
Preoperative	5.09 ± 1.21	5.36 ± 1.19	-0.85	0.39
Postoperative 3 days	1.35 ± 0.85	0.61 ± 0.88	3.24	0.002
12-month follow-up	1.26 ± 0.84	0.62 ± 0.82	2.91	0.005
Δ	3.75 ± 0.92	4.74 ± 0.96	-0.41	<0.0001
F	103.85	213.94		
*P*	<0.0001	<0.0001		
**Pfirrmann Grading(L3-4)**				
Grade I	0	0	0.46	0.795
Grade II	0	0		
Grade III	5	4		
Grade IV	19	21		
Grade V	4	6		
**Pfirrmann Grading(L5-S1)**				
Grade I	0	0	0.58	0.747
Grade II	0	0		
Grade II	2	1		
Grade III	18	22		
Grade IV	8	8		
**Pfirrmann grade of the L3–4 intervertebral disc in the UBE-PLIF group**				
	Preoperative	Postoperative		
Grade I	0	0	3.49	0.322
Grade II	3	0		
Grade III	6	5		
Grade IV	16	19		
Grade V	3	4		
**Pfirrmann grade of the L5-S1 intervertebral disc in the UBE-PLIF group**				
	Preoperative	Postoperative		
Grade I	0	0	0.95	0.621
Grade II	0	0		
Grade III	4	2		
Grade IV	18	18		
Grade V	6	8		
**Pfirrmann grade of the L3–4 intervertebral disc in the PLIF group**				
	Preoperative	Postoperative		
Grade I	0	0	2.9	0.406
Grade II	2	0		
Grade III	6	4		
Grade IV	19	21		
Grade V	4	6		
**Pfirrmann grade of the L5–S1 intervertebral disc in the PLIF group**				
Grade I	0	0	1.09	0.58
Grade II	0	0		
Grade III	3	1		
Grade IV	21	22		
Grade V	7	8		

DH: Disc Height. LL: Lumbar Lordosis. SLL: Segmental Lumbar Lordosis. SSD: Sagittal Slip Distance. Δ: Difference between postoperative day 3 and preoperative values

### Complications

**Fig. 5:**
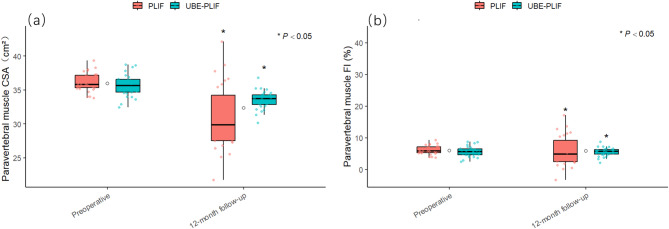
Comparison of Paraspinal Muscle-Related Parameters Between the Two Groups Before and After Surgery CSA: Cross-sectional area.FI: Fat infiltration. (a–b) Comparisons of paraspinal muscle cross-sectional area and fat infiltration between the two groups at baseline and final follow-up

The complication rate was 7.14% (2/28) in the UBE-PLIF group and 12.9% (4/31) in the PLIF group, with no statistically significant difference between the two groups (*p* = 0.465). In the UBE-PLIF group, complications included one case of dural tear and one case of epidural hematoma. In the PLIF group, two patients experienced intraoperative dural tears, one developed a postoperative wound infection, and one had hardware loosening. All six patients received appropriate symptomatic treatment, and all recovered without serious sequelae. Detailed data are shown in Fig. 5; Table 4.

**Table 4:** Comparison of preoperative and final Follow-up data between the two groups

	UBE-PLIF(*n* = 28)	PLIF(*n* = 31)	t-value/χ2	*p*-value
**Paravertebral muscle CSA(cm²)**				
Preoperative	35.75 ± 1.68	35.84 ± 1.61	-0.19	0.848
12-month follow-up	33.68 ± 1.53	30.58 ± 5.29	2.99	0.004
*P*	< 0.0001	<0.0001		
**Paravertebral muscle FI(%)**				
Preoperative	16.13 ± 1.18	16.08 ± 1.23	0.14	0.887
12-month follow-up	20.35 ± 1.72	22.73 ± 2.81	-3.86	<0.0001
*P*	<0.0001	<0.0001		
**Fusion Rate**				
Grade I	18	17	0.767	0.682
Grade II	8	10		
Grade III	2	4		
Grade IV	0	0		
**Complication Type**				
Dural tear	1	2	0.53	0.465
Epidural hematoma	1	0		
Postoperative infection	0	1		
Cage loosening	0	1		
Total complications	2(7.14%)	4(12.9%)		

CSA: Cross-sectional area.FI: Fat infiltration

## Discussion

Due to its anatomical mobility and mechanical load-bearing role, the L4–5 segment is highly susceptible to degenerative spondylolisthesis, which results from chronic axial loading and shear forces leading to disc dehydration, endplate fissures, ligamentum flavum hypertrophy, facet degeneration, and capsular laxity, ultimately compromising segmental stability and inducing vertebral slippage [11, 12]. In cases with spondylolysis or congenital posterior column dysplasia, structural weakness is further exacerbated. Loss of physiological lordosis at L4–5 alters sagittal stress distribution, accelerating adjacent segment degeneration [1]. Surgical treatment, therefore, must not only decompress neural elements but also restore segmental stability and sagittal alignment. While PLIF effectively achieves these goals, its extensive soft tissue dissection increases surgical trauma and complication risk. With the advancement of endoscopic techniques, UBE-PLIF enables sufficient decompression and stable fusion via intermuscular pathways under direct visualization, preserving posterior structures and reducing invasiveness, thereby offering a promising alternative to traditional open procedures [13, 14].

Radiological parameters are widely recognized as key indicators for assessing the quality of vertebral reduction and mechanical reconstruction. Disc height (DH), L4-5 segmental lumbar lordosis (SLL), lumbar lordosis (LL), and sagittal slip distance (SSD) directly reflect the extent of vertebral realignment and sagittal balance restoration. In this study, both UBE-PLIF and PLIF significantly improved these parameters postoperatively; however, the PLIF group demonstrated more pronounced increases in SLL, LL, SSD, and DH, likely attributable to effective mechanical correction achieved through central cage placement, bilateral distraction, and rigid screw–rod fixation [1]. Nonetheless, excessive reduction may result in uneven endplate loading, increasing the risk of cage subsidence and endplate damage. In contrast, UBE-PLIF emphasizes a more physiologic, gentle reduction under endoscopic visualization, which minimizes stress concentration on the endplates, leading to better DH preservation and a lower incidence of endplate collapse [15]. Previous studies have shown that sustained DH is critical for maintaining intervertebral foramen height and neural canal patency, both of which are essential for the long-term relief of neurological symptoms [16, 17]. Although both surgical techniques are effective in improving radiological outcomes, their fundamentally different reduction strategies and endplate protection mechanisms may have important implications for long-term postoperative structural stability.

Differences in fusion grade distribution further highlight the fundamental distinctions between UBE-PLIF and PLIF in reconstructing segmental stability. Although the UBE-PLIF group showed a higher proportion of Grade I fusion compared to the PLIF group (64.3% vs. 54.8%), the difference was not statistically significant (*p* = 0.682); nonetheless, this trend suggests that UBE-PLIF may offer certain advantages in achieving stable fusion. The quality of spinal fusion not only determines segmental mobility control but is also closely associated with postoperative pain relief, functional recovery, and the likelihood of reoperation. UBE-PLIF offers technical advantages in endplate preservation, precise autograft placement, and optimal cage positioning, which may provide a more favourable biological foundation for successful fusion. Kim et al. also reported satisfactory fusion outcomes with UBE-PLIF in patients with lumbar spondylolisthesis [18]. Benefiting from direct endoscopic visualization, this technique enables precise cartilage endplate removal while preserving structural integrity, thus promoting bone bridge formation in an ideal scaffold environment [4]. Additionally, the minimally invasive nature of UBE-PLIF reduces tissue trauma, limits local inflammatory response, and improves regional blood supply, all of which facilitate bone regeneration and integration. In contrast, high-pressure cage insertion in PLIF is more likely to damage endplates, potentially impairing bone integration and increasing the risk of postoperative cage subsidence, pseudarthrosis, and chronic low back pain [19–21]. Prior studies have also emphasized that poor fusion or endplate violation is strongly correlated with these complications and constitutes a key factor affecting long-term outcomes [22]. Although no statistically significant difference in fusion rates was observed between the two groups, the aforementioned technical differences may have important implications for the long-term prevention of segmental instability and structural failure, warranting further investigation.

Improved fusion rates often depend on a safe and controlled intraoperative environment, making the ability to manage complications a key criterion for evaluating surgical quality. Although no statistically significant difference in overall complication rates was observed between the UBE-PLIF and PLIF groups in this study, UBE-PLIF demonstrated superior safety in terms of the nature of complications and postoperative recovery. Its minimally invasive approach avoids extensive dissection and traction of deep muscle and bony structures, while the combination of high-definition magnified endoscopic views and continuous saline irrigation significantly enhances the visualization and operative precision of critical anatomical structures, including blood vessels and nerves [23, 24]. In contrast, PLIF requires wide exposure of the lamina, facet joints, and dural sac, which increases the risk of excessive nerve root manipulation, thereby elevating the likelihood of dural tears, intraoperative bleeding, and postoperative infection [25]. Therefore, while ensuring therapeutic efficacy, UBE-PLIF reduces intraoperative risks and postoperative intervention burdens through optimized surgical access and anatomical preservation, providing important advantages for accelerated recovery and sustained clinical outcomes.

This trauma control capability is reflected not only in the reduction of complications but also in its direct promotion of postoperative functional recovery and quality-of-life improvement. Based on evaluations using multiple clinical scales including VAS, ODI, and SF-36, this study found that the UBE-PLIF group exhibited significantly greater pain relief and functional improvement at one month postoperatively compared to the PLIF group, suggesting that postoperative low back pain is closely associated with muscle denervation and atrophy, thereby highlighting the importance of minimizing muscle damage during surgery [26]. This advantage may be attributed to the UBE technique’s ability to preserve the anatomical integrity and neural pathways of deep paraspinal muscles such as the multifidus and erector spinae, thus reducing the risk of myogenic pain and functional impairment [27]. Additionally, the minimally invasive nature of UBE-PLIF—characterized by less intraoperative trauma, reduced drainage, shorter hospitalization, and lower analgesic use—facilitates earlier ambulation and participation in rehabilitation [28, 29], which translates into superior outcomes in quality-of-life measures such as the SF-36. Although differences in VAS and ODI scores between the two groups diminished by the final follow-up, suggesting comparable long-term functional recovery, the significant advantage of UBE-PLIF in early postoperative recovery remains clinically meaningful. Previous MRI studies have shown that UBE can effectively reduce postoperative muscle fatty degeneration, tone loss, and perfusion deficits [30]. In contrast, traditional open procedures, due to extensive muscle stripping and thermal injury from electrocautery, often lead to impaired perfusion, inflammatory exudation, and diminished functional endurance. By utilizing a natural intermuscular plane, UBE minimizes structural muscle disruption, thereby enabling faster postoperative recovery and better early functional outcomes.

Postoperative changes in paravertebral muscle morphology—particularly reductions in paravertebral muscle cross-sectional area (CSA) and increases in fat infiltration (FI)—have become key indicators for evaluating the muscle-preserving potential and functional recovery capacity of spinal surgical techniques. In this study, the UBE-PLIF group exhibited superior preservation of paravertebral muscle CSA and better control of FI compared to the PLIF group, suggesting that its minimally invasive approach effectively reduces muscle retraction and neural irritation. In contrast, the PLIF technique, which involves extensive muscle dissection, electrocautery-induced thermal injury, and sustained traction, is more likely to compromise muscle perfusion, leading to significant paravertebral muscle CSA reduction and increased FI. Given the role of paravertebral muscles as essential stabilizers of the spine, damage to their structural integrity not only impairs functional recovery but may also accelerate adjacent segment degeneration (ASD) [31]. Although no significant differences in adjacent segment Pfirrmann grading were observed during the follow-up period, UBE-PLIF’s advantages in preserving muscular and ligamentous structures, along with its gentler restoration of sagittal alignment, may contribute to better long-term control of ASD risk. The strategy of “structural preservation plus fusion stability” is considered ideal for minimizing ASD and is highly consistent with the principles of the UBE technique. Recent studies have further emphasized that ASD is influenced not only by fusion stiffness but also by the technique’s ability to modulate sagittal alignment and protect soft tissues [32, 33]. Therefore, surgical decision-making should integrate individual anatomical, physiological, and functional considerations, aiming not only to ensure fusion stability but also to preserve muscular function and biomechanical alignment—thereby advancing spinal surgery from a model of “mechanical fusion” toward a modern paradigm that balances structure with function.

In summary, both UBE-PLIF and PLIF are effective surgical options for treating L4–5 degenerative lumbar spondylolisthesis; however, their underlying surgical philosophies differ substantially. While PLIF focuses on rigid mechanical correction through extensive exposure and forceful reduction, UBE-PLIF emphasizes anatomical preservation, physiologic alignment, and minimally invasive intervention. The findings of this study support the growing view that surgical success in spine surgery should not rely solely on radiographic fusion but also consider muscle integrity, functional recovery, complication control, and patient quality of life. The UBE-PLIF technique—by combining stable interbody fusion with soft tissue protection and sagittal alignment restoration—represents a shift from the traditional paradigm of “mechanical fusion” toward a more balanced concept that integrates biomechanical reconstruction with functional preservation. As spinal surgery continues to evolve, future research should focus not only on improving long-term fusion and stability but also on optimizing individualized surgical strategies based on anatomy, functional goals, and patient-specific needs.

## Conclusion

Both UBE-PLIF and PLIF demonstrate clear efficacy in the treatment of L4–5 degenerative lumbar spondylolisthesis. However, UBE-PLIF offers notable advantages in minimally invasive performance and rehabilitation potential, particularly in terms of reduced intraoperative trauma, improved postoperative pain relief, enhanced muscle preservation, and favourable fusion trends. By utilizing precise endoscopic manipulation and natural anatomical corridors, UBE-PLIF minimizes inflammatory responses, optimizes cage positioning and endplate preservation, and facilitates stable fusion and faster functional recovery. Across multiple clinical and radiological parameters, UBE-PLIF has demonstrated outcomes comparable to, and in some respects superior to, traditional PLIF. Given its reduced surgical burden and accelerated postoperative rehabilitation, UBE-PLIF may be particularly suitable for elderly patients or those with higher perioperative risk. In the future, this technique is expected to play an increasingly important role in the standardized and individualized management of degenerative lumbar disorders.

## Limitations

This study has several limitations. First, the relatively small sample size may have limited the statistical power of certain subgroup analyses; future multi-centre studies with larger cohorts are warranted to validate these findings. Second, as a retrospective study, it is subject to inherent selection bias and may be influenced by variations in surgical technique and surgeon experience. Prospective, randomized controlled trials are needed to establish causal relationships more robustly. Third, the follow-up duration was relatively short, precluding a comprehensive assessment of adjacent segment degeneration and long-term fusion stability. In addition, although this study integrated clinical and radiological outcomes, it did not include subjective metrics such as postoperative psychological well-being or life satisfaction, nor did it incorporate objective laboratory indicators or inflammatory markers. The learning curve associated with UBE-PLIF and cost-effectiveness analysis were also not systematically evaluated. Future studies should adopt a more comprehensive, multidimensional approach to optimize technique selection and promote personalized surgical planning.

## Data Availability

The datasets generated during and/or analyzed during the current study are available from the corresponding author on reasonable request.

## References

[1] Okpala FO. Lumbar Lordotic change and its fulcrum in low back pain disorders: radiographic evaluation. Niger J Clin Pract. 2020;23(11):1530–5. 33221777 10.4103/njcp.njcp_522_19

[2] Tiangiang Q, Renhua Q, Baozheng P, Banglei P, Deyong C, Fuguo C, et al. A comparative study on the treatment of degenerative lumbar spondylolisthesis by oblique interbody fusion and minimally invasive transforminal lumbar interbody fusion. Chin J Orthop. 2020;40(8):526–35.

[3] Bin W, Peng H, Zhenfang W, Bin X. A Meta-analysis of unilateral biportal endoscopic and micro endoscopic surgery in the treatment of lumbar spinal stenosis. Chin J Spine Spinal. 2021;31(8):719–30.

[4] Kim JE, Choi DJ. Biportal endoscopic transforaminal lumbar interbody fusion with arthroscopy. Clin Orthop Surg. 2018;10(2):248–52. 29854350 10.4055/cios.2018.10.2.248 PMC5964275

[5] Choi DJ, Kim JE. Efficacy of biportal endoscopic spine surgery for lumbar spinal stenosis. Clin Orthop Surg. 2019;11(1):82–8. 30838111 10.4055/cios.2019.11.1.82 PMC6389528

[6] Kim JE, Choi DJ, Kim MC, Park EJ. Risk factors of postoperative spinal epidural hematoma after biportal endoscopic spinal surgery. World Neurosurg. 2019;129:e324–9. 31158548 10.1016/j.wneu.2019.05.141

[7] Kang T, Park SY, Lee SH, Park JH, Suh SW. Spinal epidural abscess successfully treated with biportal endoscopic spinal surgery. Medicine. 2019;98(50):e18231. 31852084 10.1097/MD.0000000000018231 PMC6922448

[8] He LM, Chen KT, Chen CM, Chang Q, Sun L, Zhang YN, et al. Comparison of percutaneous endoscopic and open posterior lumbar interbody fusion for the treatment of single-segmental lumbar degenerative diseases. BMC Musculoskelet Disord. 2022;23(1):329. 35392878 10.1186/s12891-022-05287-9 PMC8988416

[9] Bridwell KH, Lenke LG, McEnery KW, Baldus C, Blanke K. Anterior fresh frozen structural allografts in the thoracic and lumbar spine. Do they work if combined with posterior fusion and instrumentation in adult patients with kyphosis or anterior column defects? Spine (Phila Pa 1976). 1995;20(12):1410–8. 7676341

[10] Park MK, Park SA, Son SK, Park WW, Choi SH. Correction to: Clinical and radiological outcomes of unilateral biportal endoscopic lumbar interbody fusion (ULIF) compared with conventional posterior lumbar interbody fusion (PLIF): 1-year follow-up. Neurosurg Rev. 2019;42(3):763–763. 31236727 10.1007/s10143-019-01131-2

[11] Akkawi I, Zmerly H. Degenerative spondylolisthesis: A narrative review. Acta Biomed. 2022;92(6):e2021313. 35075090 10.23750/abm.v92i6.10526 PMC8823594

[12] Tang L, Wu Y, Jing D, Xu Y, Wang C, Pan J. A bayesian network meta-analysis of 5 different fusion surgical procedures for the treatment of lumbar spondylolisthesis. Medicine. 2020;99(14):e19639. 32243393 10.1097/MD.0000000000019639 PMC7440103

[13] Fujibayashi S, Kawakami N, Asazuma T, Ito M, Mizutani J, Nagashima H, et al. Complications associated with lateral interbody fusion: nationwide survey of 2998 cases during the first 2 years of its use in Japan. Spine (Phila Pa 1976). 2017;42(19):1478–84. 28252557 10.1097/BRS.0000000000002139

[14] Bose B, Wierzbowski LR, Sestokas AK. Neurophysiologic monitoring of spinal nerve root function during instrumented posterior lumbar spine surgery. Spine (Phila Pa 1976). 2002;27(13):1444–50. 12131744 10.1097/00007632-200207010-00014

[15] Heo DH, Hong YH, Lee DC, Chung HJ, Park CK. Technique of biportal endoscopic transforaminal lumbar interbody fusion. Neurospine. 2020;17(Suppl 1):S129–37. 32746526 10.14245/ns.2040178.089 PMC7410385

[16] Santoni BG, Alexander GE, Nayak A, Cabezas A, Marulanda GA, Murtagh R, et al. Effects on inadvertent endplate fracture following lateral cage placement on range of motion and indirect spine decompression in lumbar spine fusion constructs: A cadaveric study. Int J Spine Surg. 2013;7:e101–8. 25694896 10.1016/j.ijsp.2013.09.001 PMC4300980

[17] You KH, Hwang JY, Hong SH, Kang MS, Park SM, Park HJ. Biportal endoscopic extraforaminal lumbar interbody fusion using a 3D-printed porous titanium cage with large footprints: technical note and preliminary results. Acta Neurochir (Wien). 2023;165(6):1435–43. 37115323 10.1007/s00701-023-05605-7

[18] Kim JE, Yoo HS, Choi DJ, Hwang JH, Park EJ, Chung S. Learning curve and clinical outcome of biportal Endoscopic-Assisted lumbar interbody fusion. Biomed Res Int. 2020;2020:8815432. 33381586 10.1155/2020/8815432 PMC7762649

[19] Hou Y, Luo Z. A study on the structural properties of the lumbar endplate: histological structure, the effect of bone density, and spinal level. Spine (Phila Pa 1976). 2009;34(12):E427–33. 19454994 10.1097/BRS.0b013e3181a2ea0a

[20] Park MK, Kim KT, Bang WS, Cho DC, Sung JK, Lee YS, et al. Risk factors for cage migration and cage retropulsion following transforaminal lumbar interbody fusion. Spine J. 2019;19(3):437–47. 30142459 10.1016/j.spinee.2018.08.007

[21] Long Z, Jiancheng Z, TianHang X, Xingxiao P, Yufei L. Advances in research on cage subsidence following lumbar interbody fusion. Chin J Reparative Reconstr Surg. 2021;35(8):1063–7. 10.7507/1002-1892.202104036 PMC8403988 34387439

[22] Falowski SM, Koga SF, Northcutt T, Garamszegi L, Leasure J, Block JE. Improving the management of patients with osteoporosis undergoing spinal fusion: the need for a bone mineral Density-Matched interbody cage. Orthop Res Rev. 2021;13:281–8. 34934366 10.2147/ORR.S339222 PMC8684416

[23] Heo DH, Son SK, Eum JH, Park CK. Fully endoscopic lumbar interbody fusion using a percutaneous unilateral biportal endoscopic technique: technical note and preliminary clinical results. Neurosurg Focus. 2017;43(2):E8. 28760038 10.3171/2017.5.FOCUS17146

[24] Goodnough LH, Koltsov J, Wang T, Xiong G, Nathan K, Cheng I. Decreased estimated blood loss in lateral trans-psoas versus anterior approach to lumbar interbody fusion for degenerative spondylolisthesis. J Spine Surg. 2019;5(2):185–93. 31380471 10.21037/jss.2019.05.08 PMC6626735

[25] Jisxin W, Weibing X, Dongfang Y, Guiqi Z. Research progress of unilateral biportal endoscopic spinal surgery. J Spinal Surg. 2020;18(6):425–9.

[26] Ao S, Zheng W, Wu J, Tang Y, Zhang C, Zhou Y, et al. Comparison of preliminary clinical outcomes between percutaneous endoscopic and minimally invasive transforaminal lumbar interbody fusion for lumbar degenerative diseases in a tertiary hospital: is percutaneous endoscopic procedure superior to MIS-TLIF? A prospective cohort study. Int J Surg. 2020;76:136–43. 32165279 10.1016/j.ijsu.2020.02.043

[27] Pranata R, Lim MA, Vania R, July J. Biportal endoscopic spinal surgery versus microscopic decompression for lumbar spinal stenosis: A systematic review and Meta-Analysis. World Neurosurg. 2020;138:e450–8. 32147545 10.1016/j.wneu.2020.02.151

[28] Li X, Liu J, Liu Z. Comparison of the results of open PLIF versus UBE PLIF in lumbar spinal stenosis: postoperative adjacent segment instability is lesser in UBE. J Orthop Surg Res. 2023;18(1):543. 37516831 10.1186/s13018-023-04038-3 PMC10386635

[29] Li Y, Gao SJ, Hu X, Lin SS. Comparison of efficacy between unilateral biportal endoscopic lumbar fusion versus minimally invasive transforaminal lumbar fusion in the treatment of lumbar degenerative diseases: A systematic review and meta-analysis. Medicine. 2023;102(34):e34705. 37653732 10.1097/MD.0000000000034705 PMC10470694

[30] Ahn JS, Lee HJ, Park EJ, Kim SB, Choi DJ, Kwon YS, et al. Multifidus muscle changes after biportal endoscopic spinal surgery: magnetic resonance imaging evaluation. World Neurosurg. 2019;130:e525–34. 31254694 10.1016/j.wneu.2019.06.148

[31] Jingtao J, Jun M, Wenjun L, Shan Z. Posterior lumbar interbody fusion plus screw implantation with posterior ligamentous complexes under microscope for lumbar degenerative disease. Chin J Tissue Eng Res. 2017;21(23):3682–7.

[32] Jun L, Jinming G, Hongsheng Y, BIn Z, Zhiming C, Guanhua X. The comparision of traditional approach and posterior paraspinal muscle pedicle screw fixation on the clinical efficacy and recovery of joint function in patients with lumbar fracture. J Trauma Surg. 2018;20(7):533–53336.

[33] Shikong G, Haoran G, Shu Q, Cunxiao L, Qixian Q. Clinical outcomes of improved PLIF with the adjacent vertebral posterior ligamentous complex. Orthop J China. 2014;22(11):990–5.

